# Clinical Lactation Studies of Lithium: A Systematic Review

**DOI:** 10.3389/fphar.2019.01005

**Published:** 2019-09-10

**Authors:** Maria Luisa Imaz, Mercè Torra, Dolors Soy, Lluïsa García-Esteve, Rocio Martin-Santos

**Affiliations:** ^1^Department of Medicine, Institute of Neuroscience, University of Barcelona (UB), Barcelona, Spain; ^2^Unit of Perinatal Mental Health, Department of Psychiatry and Psychology, Hospital Clínic, Institut d´Investigació Mèdica August Pi i Sunyer (IDIBAPS), Barcelona, Spain; ^3^Pharmacology and Toxicology Laboratory, Biochemistry and Molecular Genetics Service, Biomedical Diagnostic Center (CBD), Hospital Clínic, IDIBAPS, University of Barcelona, Barcelona, Spain; ^4^ Division of Medicines, Hospital Clínic, IDIBAPS, Department of Medicine, University of Barcelona, Barcelona, Spain; ^5^ Department of Psychiatry and Psychology, Hospital Clínic, IDIBAPS, Centro de Investigación Biomédica en Red en Salud Mental (CIBERSAM), Barcelona, Spain

**Keywords:** lithium, lactation, breastfeeding, human milk, postpartum, neonates, nursing infants, systematic review

## Abstract

**Background:** There is substantial evidence that postpartum prophylaxis with lithium lowers the rate of relapse in bipolar disorder. However, it is contraindicated during breastfeeding due to the high variability of the transfer into breast milk.

**Aims:** We conducted a systematic review of the current evidence of studies assessing the transfer of lithium to lactating infants and short-term infant outcomes.

**Methods:** An *a priori* protocol was designed based on PRISMA guidelines. Searches in PubMed and LactMed were conducted until September 2018. Studies assessing lithium pharmacokinetic parameters and short-term infant outcomes were included. Quality was assessed using a checklist based on international guidelines (i.e., FDA).

**Results:** From 344 initial studies, 13 case reports/series with 39 mother–child dyads were included. Only 15% of studies complied with ≥50% of the items on the quality assessment checklist. Infants breastfeed a mean (SD) of 58.9 (83.3) days. Mean maternal lithium dose was 904 (293) mg/day, corresponding lithium plasma/serum concentration was 0.73(0.26) mEq/L, and breast milk concentration was 0.84(0.14) mEq/L. Mean infant lithium plasma/serum concentration was 0.23(0.26) mEq/L. Twenty-six (80%) infants had concentrations ≤0.30 mEq/L without adverse effects. Eight (20%) showed a transient adverse event (i.e., acute toxicity or thyroid alterations). All of them were also prenatally exposed to lithium monotherapy or polytherapy.

**Conclusion:** The current evidence comes from studies with a degree of heterogeneity and of low-moderate quality. However, it identifies areas of improvement for future clinical lactation studies of lithium and provides support for some clinical recommendations.

## Introduction

Over the past decades, evidence of the health advantages of breastfeeding for neonates/infant and mothers has continued to increase, and many recommendations for practice have been published. Currently, professional organizations including The American Academy of Pediatrics (AAP, 2012), The American College of Obstetricians and Gynecologists (ACOG, 2013), and the World Health Organization (WHO, 2017) recommend breastfeeding exclusively for the first 6 months of life whenever possible, followed by combining breast milk with adequate complementary foods until the infant is 1–2 years old or beyond (Victora et al., 2016). Advantages in newborn and infants include a reduced risk of infections such as otitis media and respiratory tract infections, sudden infant death syndrome, atopic dermatitis, inflammatory bowel disease, type 1 and 2 diabetes mellitus, leukemia, and obesity (AAP, 2012; Bartick et al., 2017). Further, breastfeeding is also associated with improved neurological development (Kramer et al., 2008) and mother–infant bonding (Britton et al., 2006). In addition, the nursing mother derives benefits from breastfeeding, such as more rapid uterine involution, decreased postpartum blood loss, fertility reduction and earlier return to pre-pregnancy weight, and also a reduced risk of breast and ovarian cancers, type 1 and 2 diabetes mellitus, and cardiovascular disease and possibly also hip fracture and osteoporosis in the postmenopausal period (AAP, 2012; Bartick et al., 2017).

Bipolar disorder is considered a severe mental disorder that usually starts in the late teens and early twenties and is characterized by episodes of mania, depression, hypomania, and mixed episodes (Leboyer et al., 2005). Studies have shown that female patients with bipolar disorder are at a high risk of symptom relapse during pregnancy (Viguera et al., 2011) and the early postpartum period (Munk-Olsen et al., 2009; Viguera et al., 2011). With regard to the postpartum risk, studies have shown that 40–70% of untreated bipolar women may experience postpartum episodes of the condition (Viguera et al., 2000). There is substantial evidence that postpartum prophylaxis with mood stabilizers lowers the rate of relapse (Steward et al., 1991; Cohen et al., 1995; Bergink et al., 2012).

Lithium remains a first-line treatment for bipolar disorder during the perinatal period, given its favorable safety profile compared to other mood stabilizers (valproate, carbamazepine) (Gentile, 2012; Khan et al., 2016). The Food and Drug Administration approved lithium treatment for manic episodes of bipolar disorder and for bipolar depression, and as maintenance treatment for bipolar patients with a history of mania (López-Muñoz et al., 2018). It is also prescribed as adjunctive treatment in major depressive disorder (Bauer et al., 2003). Lithium appears to reduce the risk of suicide in patients with bipolar disorder (Cipriani et al., 2013), and it has been shown to be effective in reducing the risk of postpartum relapse (Bergink et al., 2015).

Lithium (Li^3+^) is the third element in the periodic table and is a monovalent cation that shares certain properties with sodium, potassium, and calcium. Its specific mechanisms of action in stabilizing mood are not yet well understood. At neuronal level, lithium reduces excitatory neurotransmission (i.e., of dopamine and glutamate) but increases inhibitory neurotransmission (i.e., of GABA). It may alter intracellular signaling through action on second messenger systems. Specifically, it inhibits inositol monophosphatase, possibly affecting neurotransmission *via* the phosphatidylinositol second messenger system, and it also reduces protein kinase C activity, possibly affecting the genomic expression associated with neurotransmission (Malhi et al., 2013).

Lithium is absorbed rapidly and completely after oral intake. Peak levels occur within 1 to 3 h with standard preparations and within 4 to 4.5 h with the slow and controlled release forms. It is not metabolized or bound to proteins. It is eliminated almost exclusively *via* the kidneys, although small amounts are also lost in sweat and feces, and 70–80% is reabsorbed primarily in the proximal tubule of the kidney. Lithium’s elimination half-life is about 18–24 h in healthy young subjects. Steady state concentrations are achieved within 4–5 days (Alda, 2006; Malhi et al., 2013). The target plasma level for lithium in acute treatment is 0.8–1.2 mEq/L in young subjects, while in maintenance treatment, the most common optimal plasma concentration range is 0.5–0.8 mEq/L (Gelenberg et al., 1989; Malhi et al., 2017; Hiemke et al., 2018).

Physiological changes during pregnancy (Feghali et al., 2015) may alter the pharmacokinetics of lithium and can cause a notable decline in maternal lithium serum concentrations during this period. In the third trimester, lithium clearance rose by 30–50% (Grandjean and Aubry, 2009; Westin et al., 2017; Wesseloo et al., 2017) because of increased plasma volume and greater glomerular filtration rate (Davison and Dunlop, 1980; Deligiannidis et al., 2014). Lithium has a complete placental passage with ion equilibration across placental barrier that is remarkably uniform across a wide range of maternal concentrations (0.2–2.6 mEq/L) (Newport et al., 2005). Its levels rise slightly in the immediate postpartum (Wesseloo et al., 2017) because the glomerular filtrate returns to pre-pregnancy levels after delivery (Davison and Dunlop, 1980; Deligiannidis et al., 2014). Use of lithium in late pregnancy may produce toxicity in the newborn: this is usually transient and reversible, but neonates may present respiratory distress syndrome, cyanosis, lethargy, depressed neonatal reflexes, hypotonia, bradycardia, and feeding difficulties (Kozma, 2005; McKnight et al., 2012). These complications are associated with lithium concentrations in cord blood above 0.64 mEq/L (Newport et al., 2005). In this situation, neonates may require supportive care for 10–14 days until they eliminate lithium. No long-term neurodevelopmental effects have been reported in infants exposed to lithium in utero (Schou, 1976; Van der Lugt et al., 2012; Poels et al., 2018).

**Figure 1:**
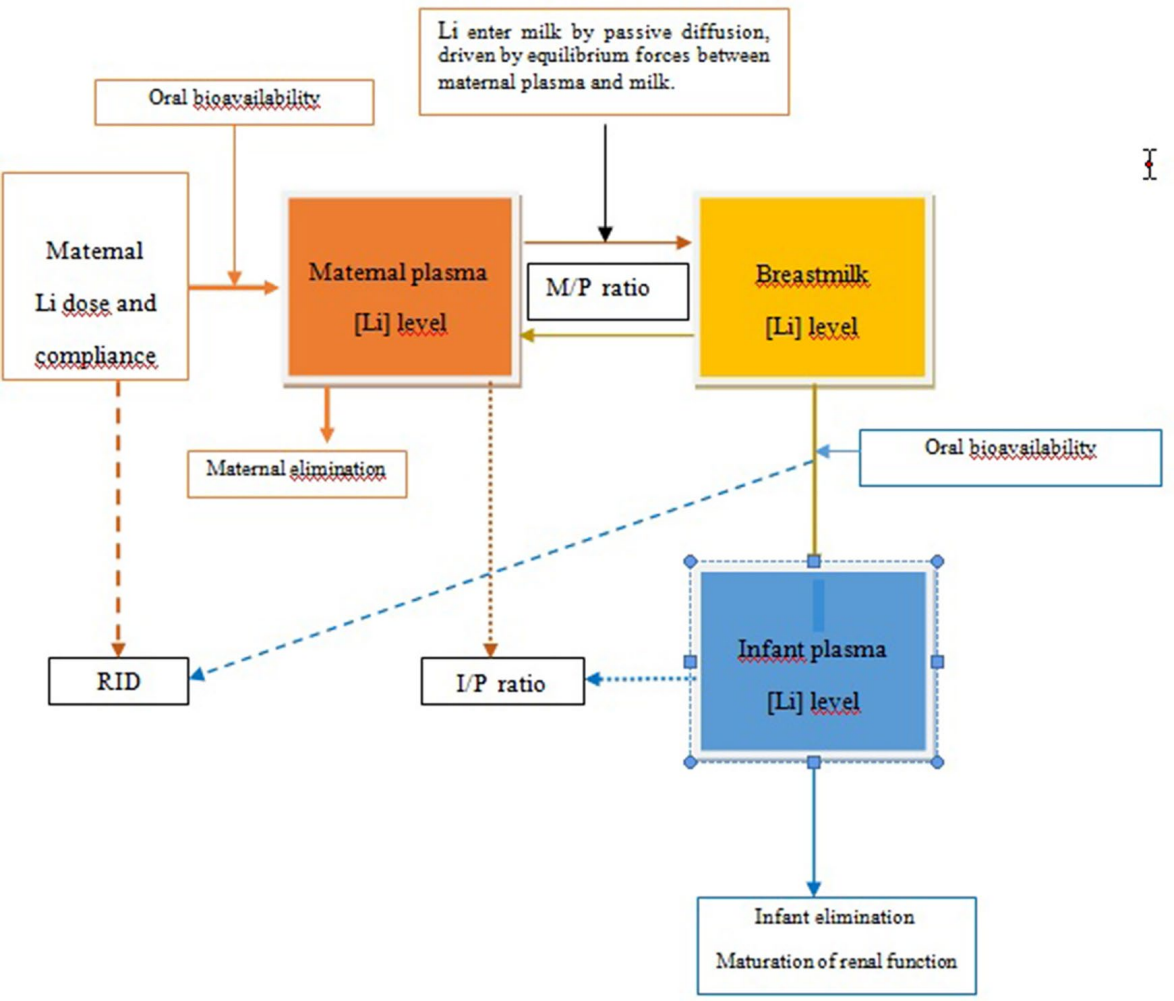
Transfer of lithium from mother to infant *via* breastmilk. I, infant plasma; M, milk; P, maternal plasma; RID, relative infant dose.

With regard to breastfeeding, lithium is excreted in human breast milk at a mean rate of approximately 50% (range 0.17–1.07%) of the mother serum concentration (Weinstein and Goldfield, 1969; Fries, 1970; Tunnessen and Hertz, 1972; Schou and Amdisen, 1973; Sykes et al., 1976; Viguera et al., 2007; Tanaka et al., 2008). First 4 to 10 days postpartum, lithium can pass between alveolar cells because large gaps exist. By the end 1st week postpartum, alveolar cells swell under influence of prolactin, closing the intracellular gaps, and limiting access to the milk (Pons et al., 1994) (**Figure 1**). Factors that affect the passage of a drug into breast milk include route of administration, absorption rate, half-life, peak serum time, dissociation constant, volume of distribution, molecular size, protein binding, degree of ionization, pH, and solubility (Lawrence, 1994; Tanaka et al., 2008). Because of its very low molecular weight and lack of protein binding, lithium is readily transferred into breast milk. The amount of drug received by the infant also depends on multiple factors: milk yield and composition (i.e., colostrum *versus* mature milk), concentration of the drug in the milk, which breast is being suckled (as the yield from each breast is not equal), and how well the breast was emptied during the previous feeding (Lawrence, 1994; Pons et al., 1994). However, the mean volume of milk transferred to the infants is lower during the first 2 days after delivery and increases rapidly on days 3 and 4, and then more slowly to a maximum of approximately 800ml/day at 6 months of age (Neville et al., 1988). An infant’s ability to absorb, detoxify, and excrete the drug are important factors (Lawrence, 1994). Less mature infants are less able to clear drugs because of their immature liver and renal functions (Pons et al., 1994), and so medications that are predominately eliminated through the kidney, such as lithium, may accumulate (Flaherty and Krenzelok, 1997). The infant’s age also affects the amount of milk consumed, since in older infants nourishment is supplemented (AAP, 2012; ACOG, 2013; Victora et al., 2016; WHO, 2017). Other factors include any medical problems that the infant may have. At present, no information on lithium exposure *via* breast milk for preterm or ill infants is available (Bogen et al., 2012).

Although lithium is contraindicated during breastfeeding in many treatment guidelines (Hirschfeld et al., 2002; National Institute for Health and Clinical Excellence (NICE), 2014; Malhi et al., 2015; McAllister et al., 2017; Yatham et al., 2018) due to the high variability of the transfer into breast milk, other sources do not argue against its use (Uguz and Sharma, 2016; Pacchiarotti et al., 2016) especially when maternal mood is stable, during lithium monotherapy (Viguera et al., 2007) and in healthy infants over 2 months of age (Anderson et al., 2003; Soussan et al., 2014; Anderson et al., 2016). Finally, other studies favor its use under strict infant clinical monitoring (Bogen et al., 2012).

In clinical practice, it is difficult to decide whether to initiate, maintain, or discontinue lithium treatment during breastfeeding. Increasingly, women with bipolar disorder are expressing a desire to breastfeed while receiving lithium (Galbally et al., 2018). Women who may benefit from lithium in the postpartum period and who want to breastfeed are encouraged to discuss these decisions with their healthcare providers (obstetricians, psychiatrists, and pediatricians) in a collaborative manner.

The aim of the present paper was to systematically review the current evidence and quality of studies assessing the transfer of lithium to lactating infants, and their short-term outcomes.

## Methods

Data for this systematic review were collected with an advance protocol (see supporting information) based on the Preferred Reporting Items for Systematic Reviews and Meta-analyses (PRISMA) guidelines (Moher et al., 2009). The protocol for this systematic review was published *via* PROSPERO (registration code CRD42019120928). Two of the authors (MLI, MT) independently reviewed all the studies retrieved, and differences in opinion were resolved by consensus, and when necessary after discussion with a third researcher (RMS) (see **Supplementary material**).

### Search Strategy

Papers published in electronic databases including PubMed and LactMed between 1 January 1995 and 28 September 2018 were sought, using the following terms: “lithium,” “lactation,” “breastfeeding,” “postpartum period,” “puerperium,” “neonates,” and “nursing infants” mixed with Boolean operator “AND.” Experimental studies involving animals, reviews or meta-analyses, letters to the editor, editorials, and commentaries were excluded. After the titles of all non-duplicated articles had been identified, the abstracts were screened to ensure that they met the inclusion criteria. Full texts of the relevant abstracts were obtained and examined carefully to determine their eligibility for inclusion. Additionally, references in the papers were examined in order to identify further relevant publications. We tried to get in contact with authors when missing data.

### Study Selection

Only articles containing primary data in humans were considered for inclusion in the systematic review, in accordance with the following predefined criteria: (1) case reports, case series, case-control studies, cohort studies, quasi-experimental, or experimental studies; (2) studies that monitored lithium concentration in mother (plasma/serum and/or breast milk) and infants (plasma/serum) during the lactation period; (3) use of clearly defined pharmacokinetic parameters such as the infant-plasma concentration and/or the milk-to-plasma ratio (M/P ratio), relative infant dose (RID), and/or the infant-plasma-to-maternal plasma ratio (I/P ratio); (4) type of concomitant medication used; (5) well-defined adverse events or developmental outcomes in the infants; and (6) studies published in English or Spanish in a peer-reviewed journal.

### Data Extraction and Main Outcomes

The variables recorded for each study were: author, year of publication, country, study design, sample size, maternal diagnosis, maternal weight, type of breastfeeding, duration of breastfeeding, medication regimen administered to the mother during pregnancy and lactation (lithium and concomitant drugs), type of delivery, gestational age, birth weight, infant sex, Apgar minutes 1–5, infant age at sampling (weeks + days), lithium plasma/serum concentrations in mother and infant, lithium milk concentrations, pharmacokinetic parameters, and neonate and infant adverse effects.

### Assessment of Pharmacokinetic Parameters (Direct and Estimated)

The amount of drug transferred to infant was measured directly in infant-plasma/serum or estimated on the basis of pharmacokinetic parameters (M/P ratio or RID) (Begg et al., 2002; FDA, 2005; Sachs and Committee on Drugs, 2013).

#### Direct Measures

##### Infant-Plasma Drug Concentration (I)

The infant plasma/serum concentration provides information regarding the fraction of drug that is systematically available to the breastfed child (Begg et al., 2002). It is the most direct measure for risk assessment (FDA, 2005; European Medicine Agency, 2009). However, in women who take lithium in late pregnancy, infant levels measured in the early neonatal period (first 7–10 days postpartum) may reflect transplacental passage of lithium rather than its intake *via* breast milk (Hale and Rowe, 2017). Another point to take into account is that this invasive exploration may be painful for the infant and may be rejected by parents.

Toxic levels of lithium in plasma or serum have not been established. The best approximation at present is the case series study of 10 mother–infant pairs in which the mother received lithium monotherapy during pregnancy and lactation (Viguera et al., 2007). In this study, no infants showed signs and symptoms of lithium toxicity, and lithium infant-plasma levels were below 0.30 meq/L.

#### Estimated Measures

##### Milk-to-Maternal Plasma Drug Concentration Ratio (M/P Ratio)

The M/P ratio is an estimate of the distribution of the drug between maternal plasma and milk. It is calculated by dividing the concentration of the drug in the mother’s milk by the concentration in the mother’s plasma. The currently accepted method for calculating the M/P ratio is to use the ratio between the area under the curve (AUC) for milk and plasma. The calculation of the AUC from the collection of several samples (five or six) of steady state lithium over a specific time interval is probably the most suitable method (Begg et al., 2002) **Figure 1**.

A M/P ratio <1 is a good indicator that only minimal levels of the drug are transferred into the milk, while a ratio >1.5 implies that high levels of the drug may be sequestered in milk (Begg et al., 2002). From a clinical perspective, the M/P ratio does not predict the safety of a drug for the child during breastfeeding (Begg et al., 2002).

##### The Relative Infant Dose (RID)

The RID is calculated by dividing the infant’s dose *via* milk in mg/kg/day by the maternal dose in mg/kg/day. This weight-normalizing method indicates approximately how much of the maternal dose the infant is receiving (**Figure 1**).

Several cutoff points have been proposed for this index (Atkinson et al., 1988). A RID <10% of the lowest end of the weight-adjusted maternal dosage is considered acceptable for breastfed infants, and RIDs >25% should be avoided in nursing mothers. Recently, a joint working group in Denmark developed guidelines for the use of psychotropic drugs during breastfeeding which used an equally arbitrary, but more conservative cutoff of 5% as the limit of breastfeeding acceptability (Larsen et al., 2015).

Although the RID is accepted as a measure of the safety of medication during breastfeeding, it has some limitations. For example, if the drug dose given to the mother increases, so does the infant’s dosage received *via* breast milk, but the RID does not usually change. Therefore, the RID is unreliable for representing drug safety during breastfeeding for a drug with a wide dosage range, especially those with an RID near the 10% cutoff point. Another limitation is that the RID does not account for the possibility of differences in bioavailability of the drug related to infant age (Anderson and Sauberan, 2016).

##### The Infant-Plasma-to-Maternal Plasma Drug Concentration Ratio (I/P Ratio)

The I/P ratio is the concentration of drug in the infant’s plasma divided by the concentration in the mother’s plasma. The plasma concentration comparison is appealing because it minimizes variables such as bioavailability and differences in clearance between the infant and mother. It is most accurate when applied at steady-state for drugs that have a relatively long elimination half-life because maternal and infant levels do not fluctuate substantially. When samples are obtained in these conditions, a reliable measurement for single trough blood samples from the mother and infant would probably suffice, although this possibility has not been rigorously tested. For drugs with a short elimination of half-life, multiple plasma samples are required to obtain average plasma concentrations or AUC measurements to derive a reliable I/P ratio (Anderson and Sauberan, 2016). In the case of lithium, the half-life is about 18 to 24h in healthy young women, but it appears to be longer in neonates—close to 96 h with a high interindividual variability (range: 1.42–36.09 days) (Guitart et al., 2013).

As with the RID, a drug that produces a steady-state I/P ratio below 10% of the lowest end of the therapeutic concentration range was considered acceptable by the American Academy of Pediatric, and a ratio above 25% was considered unacceptable (Sachs and Committee on Drugs, 2013). A problem with the I/P ratio in relation to the time of infant sampling may occur if the mother was taking the drug during pregnancy. In general, a much larger amount of the drug is passed to the fetus transplacentally than to the infant *via* breast milk. Therefore, obtaining infant blood samples too soon after delivery (<7 days) may reflect transplacental passage rather than breast milk transfer (Hale and Rowe, 2017).

### Quality Assessment

The quality review of all studies was based on the guidelines of the International Lactation Consultant Association, the Food and Drug Administration, and the European Medicine Agency (Begg et al., 2002; FDA, 2005; European Medicine Agency, 2009). These guidelines provide recommendations for conducting clinical lactation studies. We recorded data on study design, clinical conduct, endpoints correctly assessed, and laboratory methods in a checklist and also added one more item: the presence or absent of adverse events.

All studies that met the criteria were assessed using this checklist. Their quality was calculated by dividing the number of items scored by the total number of items and recorded as a percentage (**Table 3**).

## Results

### Study Selection

**Figure 2:**
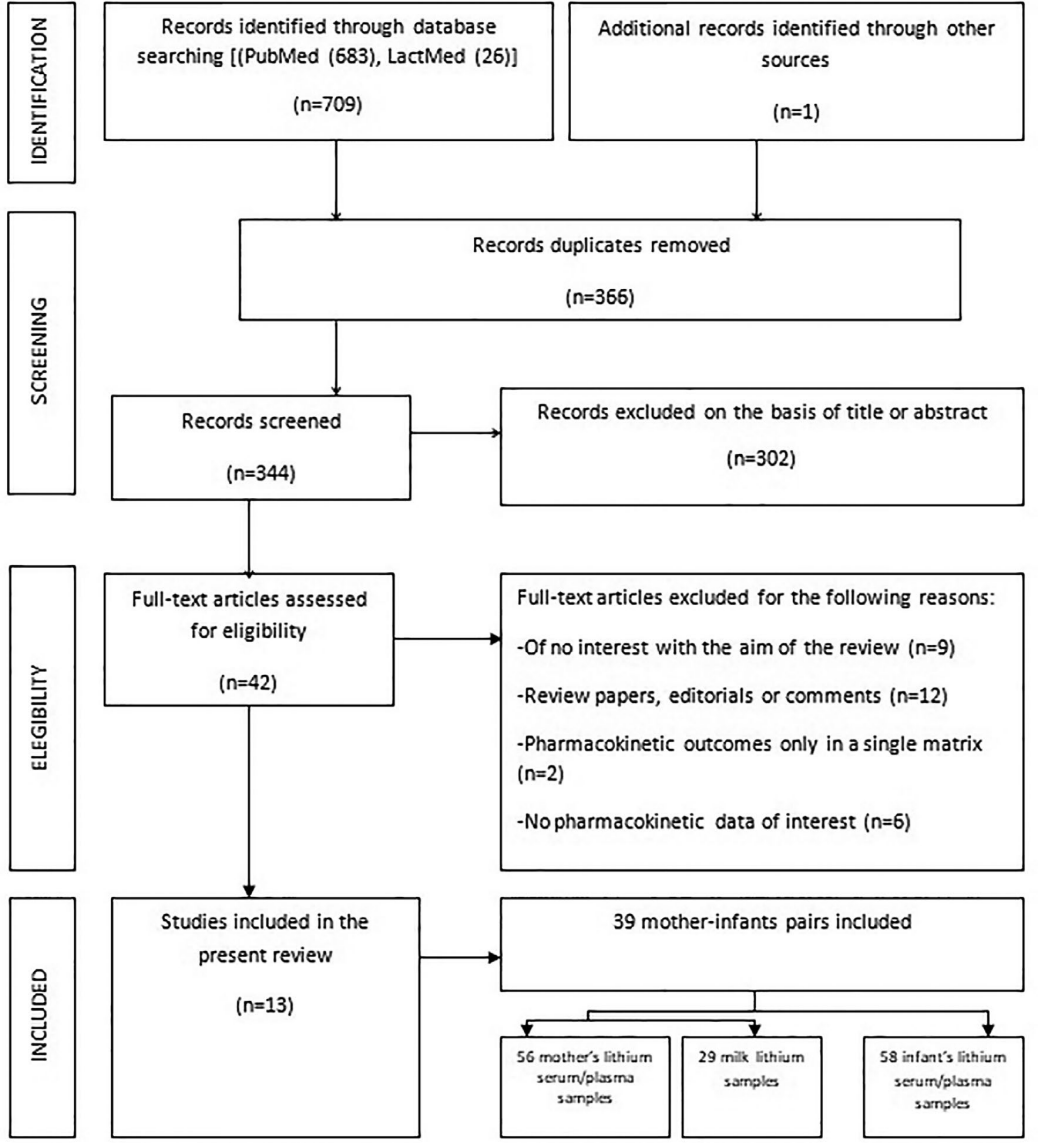
Flowchart of considered and finally selected studies, according to the PRISMA statements.

Of 709 records, 366 were removed after screening due to duplication, and 302 were excluded after title/abstract review because they did not meet the selection criteria *a priori*. Forty two full texts were then assessed for eligibility. Of these, 29 articles were excluded for reasons shown in **Figure 2**. Articles were selected in accordance with the PRISMA statement, and the process is outlined in **Figure 2**.

### Description of Studies

Thirteen studies—9 case reports (Weinstein and Goldfield, 1969; Fries, 1970; Tunnessen and Hertz, 1972; Sykes et al., 1976; Montgomery, 1997; Skausing and Shou, 1977; Tanaka et al., 2008; Marín et al., 2011; Frew, 2015) and 4 case series (Schou and Amdisen, 1973; Moretti et al., 2003; Viguera et al., 2007; Bogen et al., 2012) including a total of 40 mothers with severe mental disorders treated with lithium during lactation were published in the literature between 1969 and 2018. Twenty-nine mothers had bipolar disorder, 1 had recurrent depressive disorder, and in 10, the condition was not specified. Thirty-nine out of 40 mother–infant pairs had at least 1 simultaneous determination of lithium in the mother (serum or milk) and in the child (serum). All articles included were written in English or Spanish with the exception of one Danish article with an informative English abstract (Skausing and Shou, 1977). Thus, the final sample comprised 13 studies including 39 mother–infant cases.

Only one study each provided information on maternal ethnicity (Bogen et al., 2012) and weight (Moretti et al., 2003). Similarly, only one study provided information on smoking or alcohol status, or concomitant illness in pregnancy or postpartum (Moretti et al., 2003). Some studies reported the gravidity/parity index (Tunnessen and Hertz, 1972; Sykes et al., 1976; Bogen et al., 2012; Frew, 2015), gestational age (Tunnessen and Hertz, 1972; Sykes et al., 1976; Moretti et al., 2003; Tanaka et al., 2008; Bogen et al., 2012; Frew, 2015), and weight birth (Tunnessen and Hertz, 1972; Sykes et al., 1976; Tanaka et al., 2008; Marín et al., 2011; Bogen et al., 2012).

From the 39 cases, 22 were treated with lithium during the index pregnancy (Weinstein and Goldfield, 1969; Fries, 1970; Tunnessen and Hertz, 1972; Sykes et al., 1976; Moretti et al., 2003; Viguera et al., 2007; Tanaka et al., 2008, Marín et al., 2011; Bogen et al., 2012; Frew, 2015), and in 17 cases, no information was provided. Moreover, four cases received polytherapy in pregnancy (Tunnessen and Hertz, 1972; Bogen et al., 2012), and 3 more during lactation (Sykes et al., 1976; Marín et al., 2011; Frew, 2015). Sixteen mothers practiced exclusive breastfeeding (Viguera et al., 2007; Marín et al., 2011; Bogen et al., 2012; Frew, 2015), and in the other cases, no information was available on the type of maternal lactation. **Table 1** summarizes the characteristics of the studies included.

**Table 1:** Characteristics of the studies included in the systematic review: maternal diagnosis, treatment during pregnancy and lactation, and obstetric and neonatal outcomes.

Author year/country	Study design, N	Mother–infant pair code_n_	Pregnancy				Postpartum					
			Mother				Neonate				Mother	
			Age (years)	Diagnosis	Medication (mg/day)	[Li] (meq/L)	Type of delivery	Gestational age (weeks)	Birth weight (g), sex	Apgar 1/5 min	Medication (mg/day)	Type of breastfeeding
Weinstein and Goldfield, 1969/Sweden	Case report N = 1	1	NA	NA	Lithium 1,000	0.33–0.35	NA	NA	NA	NA	Lithium 1,000	Not specified
Fries, 1970/Sweden	Case report N = 1	2	NA	NA	Lithium 900	0.90	NA	NA	NA	NA	Lithium 900	Not specified
Tunnessen and Hertz, 1972/USA	Case report N = 1	3	31	RDD	Lithium 600–1,200^b^ others^c^	1.07–1.38	Vaginal instrumented	38	2,910/F	6/9	Lithium	Not specified
Schou and Amdisen, 1973/Denmark	Case series N = 5	4–8	NA	NA	NA	NA	NA	NA	NA	NA	Lithium	Not specified
Sykes et al., 1976/UK	Case report N = 1	9	36	BD	Lithium 800–400	0.61–1.20 D: MP 0.32-UC 0.32	Vaginal instrumented	38	3,450/M	NA	Lithium: 400–800 others^e^	Not specified
Skausing and Schou, 1977/Denmark	Case report N = 1	10	NA	NA	NA	NA	NA	NA	NA	NA	Lithium	Not specified
Montgomery, 1997/USA	Case report N = 1	11	NA	NA	NA	NA	NA	NA	NA	NA	Lithium 900	Not specified
Moretti et al., 2003/Canada	Case series N = 11	12–22	NA	BD	Lithium	NA UC 0.43 (C_18_)	NA	NA 32 wk (C_20_)	NA	NA	Lithium 600–1,500 From month 15(C_22_)	Not specified Mixed 50/50 (C_20_)
Viguera et al., 2007/USA	Case series N = 10	23–32	NA	BD	Lithium 34wk-D (C_28_)	NA	NA	NA	NA 1 M/9 NA	NA	Lithium 600–1,200	Exclusive
Tanaka et al., 2008/Canada	Case report N = 2	33	32	BD	Lithium 1,500	0.34–0.48	NA	31	1,700/M	7/8	Lithium 1,200	Not specified initiated day 7
		34^a^	37	NA	Lithium 900	0.9 at delivery	NA	Full term	3,100/F	7/8	NA	Not specified
Marín et al., 2011/Spain	Case report N = 1	35	NA	BD	Lithium 800	NA	Caesarean section	40	3,140/M	9/10	Lithium 800 + others^e^	Exclusive
Bogen et al., 2012/USA	Case series N = 4	36	28	BD type I	Lithium 900 + others^c^	0.20–0.55 0.48 (−30 days)^d^	NA	40	3,405/M	8/9	Lithium 900	Exclusive
		37	30	BD type I	Lithium 900 + others^c^	0.60 (−14 days)^d^	NA	41	4,026/F	9/9	Lithium 900	
		38	19	BD type I	Lithium 600–900	0.40 (−5 days)^d^	NA	38	4,045/M	9/9	Lithium 900	
		39	28	BD type I factor V Leiden	Lithium + others^c^	0.85(−180 days)^d^	NA	38	3,501/F	N/K	Lithium 1,500 alt 1,200	
Frew, 2015/USA	Case report N = 1	40	34	BD type I	Lithium 600–900 TM 2–3	NA	NA	Full term	NA	NA	Lithium 600 + others^e^	Exclusive

ALT, alternate; BD, bipolar disorder; D, delivery; F, female; M, male; MP, mother plasma; NA, not available; NK, not known; RDD, recurrent depressive disorder; TM, trimester; UC, umbilical cord; WK, week.

^a^Excluded case: did not meet inclusion criteria of the review; ^b^Estimated data through the graph; ^c^Medication other than lithium during pregnancy: C_3_: chlorthalidone 50 mg every three days for the last four months of pregnancy, thyroglobulin 90 mg/day, secobarbital 30 mg/day occasionally, chloramphenicol and iodochlorhydroxyquin during the second trimester; C_36_: Bupropion 300 mg, Levothyroxine 50 µg, prenatal vitamins, calcium, fish oil; CN_37_: Bupropion 300 mg, Levothyroxine 75 µg, prenatal vitamins, fish oil, iron, vitamin D; C_39_: escitalopram 10 mg, synthroid 25 µg, heparin docusate 200 mg, polyethylene glycol, prenatal vitamines, fish oil, magnesium 800 mg; ^d^Day before delivery; ^e^Medication other than lithium during postpartum: CN_9_: Pethidine HCI 100 + Promazin HCI 50 6 h before delivery; C_35_: Olanzapine 2.5 mg days 0-15; C_40_: Quetiapine, Aripiprazol 2.5 two weeks.

Gravidity-Parity Index Case: CN3: G2P2; CN 9: G1P0; CN 36: G2P1; CN 37: G3P2; CN 38: G1P0; CN39: G3P0; CN 40: G2P2.

### Pharmacokinetic Results

The pharmacokinetic results (mean, SD, and range) are shown in **Table 2**. The 39 mother–infant pairs contributed 56 maternal serum samples, 29 maternal milk samples, and 58 infant serum samples. The samples were obtained in a very wide range between 1 day and 385 days postpartum. Infants were breastfed an average of 58.86 (83.25) days (SD), but in most studies, only one or a few measurements were recorded. The maternal daily dose of lithium was between 400 and 1,500 mg/day, with a mean (SD) of 904 (283) mg/day and corresponding mean (SD) lithium serum concentrations o 0.73 (0.26) mEq/L (0.12–1.50).

**Table 2:** Data of simultaneous monitorization of lithium concentration in mother (serum and/or breast milk) and infant (serum) during breastfeeding and infant outcomes.

Author, year	Mother–infant pair code	Infant age at sampling (week+days)	Maternal weight (Kg)	Maternal lithium dose (mg/day)	[Li] (mEq/L) serum/breast milk			Pharmacokinetic parameters				Infant adverse effects/duration breastfeeding
					Mother (P) N = 56	Breast milk (M) N = 29	Infant (I) N = 58	M/P ratio N = 28	RID (%) N = 23	I/M ratio N = 25	I/P ratio N = 47	
Weinstein and Goldfield, 1969	1	2+3	NA	1,000	0.84	NA	0.04	–	–	–	0.04	None/NA
		10	NA	1,000	0.50	0.12	NA	0.24	–	–	–	
Fries, 1970	2	1	NA	900	0.90	0.30	0.30	0.33	–	1	0.33	None/NA
Tunnessen and Hertz, 1972	3	0+5	NA	NA	1.5	0.60	0.60	0.40	–	1	0.40	Transient lithium toxicity. The baby was normal by day 8/stop at 5 day
		1	NA	NA	NA	NA	0.21	–	–	–	–	
Schou and Amdisen, 1973	4	1	NA	NA	0.34	0.16	0.22	0.47	–	1.37	0.65	NA/NA
	5	2	NA	NA	0.90	0.30	0.30	0.33	–	1	0.33	NA/NA
	6	2	NA	NA	0.84	0.56	0.15	0.66	–	0.27	0.17	NA/NA
	7	3^c^	NA	NA	0.57	0.24	NA	0.42	–	–	–	NA/NA
	8	4	NA	NA	NA	0.50	0.10	–	–	0.20	–	NA/NA
Sykes et al., 1976	9	0+6	NA	400	0.35	0.20	0.03	0.57	–	0.15	0.08	Mildly hypotonic for the first 2 days. Over 63 days showed no negative effects/started within 6 days and stop on week 10
		1	NA	400	0.27	0.14	0.09	0.51	–	0.64	0.33	
		2	NA	600	0.69	0.27	0.06	0.39	–	0.22	0.08	
		4	NA	800	0.95	0.69	0.12	0.72	–	0.17	0.12	
		6	NA	800	1.10	0.27	0.10	0.24	–	0.37	0.09	
		9	NA	800	0.89	0.25	0.09	0.28	–	0.36	0.10	
Skausing and Shou, 1977	10	8	NA	NA	0.70	NA	1.40	–	–	–	2.00	Upper respiratory infection and probably dehydration of 2 months. Lithium toxicity recovered after stop breastfeeding/stop 2 months
Montgomery, 1997	11	2	NA	900	0.62	–	0.31	–			0.50	None. Neurobehavioral and thyroid normal/NA
		4	NA	–	–	–	0.29	–	–	–	–	
Moretti et al., 2003	12	0+1	65.9	600	NA	NA	NA	–	3.50	–	–	None/NA
		0+2	65.9	600	NA	NA	NA	–	3.50	–	–	
		0+4	65.9	900	NA	NA	NA	–	19.90	–	–	
		2	65.9	900	NA	NA	NA	–	19.90	–	–	
	13	2	60	600	NA	NA	NA	–	5.50	–	–	NA/NA
	14	0+5	84	1,500	0.70–0.80	NA	0.14	–	21	–	0.20–0.17	None/NA
		8	84	1,500	0.90	NA	0.22	–	30	–	0.24	
	15	3	NA	900	NA	NA	NA	–	15.50	–	–	None/NA
		3+2	NA	900	NA	NA	NA	–	15.50	–	–	
	16	0+2	67	900	NA	NA	NA	–	24.70	–	–	None/NA
		0+3	67	900	NA	NA	NA	–	15	–	–	
		1+4	67	900	NA	NA	NA	–	25	–	–	
		3+4	67	900	0.47	NA	0.47	–	–	–	1	
	17	1+2	83	600	NA	NA	NA	–	f	–	–	None/NA
		6	83	600	NA	NA	NA	–	f	–	–	
		32	83	600	NA	NA	NA	–	f	–	–	
	18	0	90	600	NA	NA	0.43	–	–	–	–	None/NA
		0+5	90	600	NA	NA	NA	–	15.00	–	–	
		3	90	600	NA	NA	NA	–	6.50	–	–	
	19	2+3	73	1,125	NA	NA	NA	–	6.80	–	–	Neonatal complication no before breastfeeding began/NA
	20	0	85	1,500	0.58	NA	NA	–	–	–	–	None/NA
		1+2	85	1,500	NA	NA	NA	–	15.70	–	–	
		4	85	1,500	1.34	NA	NA	–	–	–	–	
		5+4	85	1,500	NA	NA	NA	–	23.00	–	–	
	21	0+2	60	1,200	NA	NA	NA	–	8.20	–	–	None/NA
		12	60	1,200	NA	NA	NA	–	5.50	–	–	
	22	>60	130	600	NA	NA	NA	–	<5.00	–	–	None. Lithium started at 15 months /NA
Viguera et al., 2007	23	7	NA	600	0.43	0.30	0.10	0.70	–	0.33	0.23	None/NA
	24	10	NA	600	0.70	0.28	0.20	0.40	–	0.28	0.28	None/NA
		21	NA	600	0.70	NA	0.22	–	–	–	0.31	
		52	NA	600	0.60	0.10	0.10	0.17	–	1	0.17	
	25	1	NA	625	0.80	NA	0.30	–	–	–	0.37	Urea nitrogen 19 mg/dl, creatinine 0.6 mg/dl. No clinical signs of hypovolemia. Normal 1 year later/NA
		8	NA	625	0.70	NA	0.30	–	–	–	0.42	
		14	NA	625	0.70	NA	0.30	–	–	–	0.42	
		24	NA	725	0.60	0.44	0.10	0.73	–	0.17	0.16	
		30	NA	725	0.60	NA	NA	–	–	–	–	
		55	NA	750	0.70	0.46	NA	0.66	–	–	–	
	26	32	NA	700	0.60	0.36	0.09	0.60	–	0.25	0.15	None/NA
	27	4	NA	900	0.90	0.39	0.30	0.43	–	0.77	0.33	None/NA
		12	NA	900	1.00	0.25	0.10	0.25	–	0.40	0.10	
	28	7	NA	900	0.41	0.25	0.23	0.61	–	0.92	0.56	TSH 7.1 µU/ml; TSH 2.07 µU/ml after stop lithium^g^/NA
	29	2	NA	900	0.80	NA	0.10	–	–	–	0.12	Urea nitrogen 22 mg/d. No clinical signs of hypovolemia. Normal 1 year later/NA
		5	NA	900	0.80	0.51	0.13	0.64	–	0.25	0.16	
		14	NA	900	0.92	0.40	0.10	0.43	–	0.25	0.10	
		32	NA	900	0.92	NA	0.20	–	–	–	0.21	
		52	NA	900	NA	NA	0.10	–	–	–	–	
	30	8	NA	900	1.31	NA	0.14	–	–	–	0.11	None/NA
	31	6	NA	1,200	1.16	0.48	0.19	0.41	–	0.40	0.16	None/NA
		25	NA	1,200	1.03	NA	0.05	–	–	–	0.04	
	32	4	NA	1,200	0.55	0.37	0.10	0.67	–	0.27	0.18	None/NA
		10	NA	1,200	0.55	NA	0.10	–	–	–	0.18	
		14	NA	1,200	0.67	0.40	0.18	0.60	–	0.27	0.26	
		25	NA	1,200	0.65	NA	0.14	–	–	–	0.21	
Tanaka et al., 2008	33	0+1	NA	1,200	0.41	0.44	4.19^e^	1.07	–	–	–	Spurious toxic infant lithium level suspected. Tube feeding and mother´s milk start on day 7
		0+4	NA	1,200	NA	NA	0.11	–	–	–	–	
		0+6	NA	1,200	NA	NA	<0.10	–	–	–	–	
		1+3	NA	1,200	NA	NA	<0.10	–	–	–	–	
	34^b^	0+3	NA	NA	NA	NA	<0.30	–	–	–	–	Spurious toxic infant lithium level suspected. None. NA
		0+6	NA	NA	NA	NA	0.70	–	–	–	–	
		1+3	NA	NA	NA	NA	1.10	–	–	–	–	
		1+4	NA	NA	0.70	NA	NA	–	–	–	–	
		2+4	NA	NA	NA	NA	1.10	–	–	–	–	
Marín et al., 2011	35	2+1	NA	800	0.74	NA	0.26	–	–	–	0.35	TSH (µU/ml): 5.14 at 1 month; 3.55 at 2 months; 2.17 at 6 months. No electrolyte or liver abnormalities. Normal neurodevelopment at 6 months/NA
		4	NA	800	NA	NA	0.23	–	–	–	–	
		8	NA	800	NA	NA	0.23	–	–	–	–	
		24	NA	800	NA	NA	0.17w	–	–	–	–	
Bogen et al., 2012	36	4+3	NA	900	0.72	NA	0.08	–	–	–	0.11	Weight loss (4.2% day 2, 5.9% day 7). Feeding problems. Mild hypotonia (2 months). Early intervention care for gross and fine motor delay through the first year/duration past 1 year
		26+1	NA	900	0.48	NA	0.08	–	–	–	0.17	
	37	6+1	NA	900	0.73	NA	0.11	–	–	–	0.15	None/NA
	38	0+4	NA	900	0.78	NA	NA	–	–	–	–	Weight loss (5.2% day 2 and 8.5% day 3). Feeding problems. 4 months
		6+3	NA	900	0.81	NA	0.08	–	–	–	0.10	
	39	2	NA	1,350 ^d^	0.12	NA	NA	–	–	–	–	None/7 months
		4+3	NA	1,350 ^d^	0.97	NA	0.11	–	–	–	0.11	
Frew, 2015	40	1+3	NA	600	0.45	NA	0.26	–	–	–	0.58	None/NA
	Mean (SD) range	8.61 (12.98) [0–60]		904.2 (283.2) [400–1,500]	0.73 (0.3) [0.12–1.50]	0.34 (0–14) [0.10–0.69]	0.23 (0.26) [0.03–1.40]	0.49 (0.19) [0.17–1.07]	12.12 (8.5) [0–30]	0.49 (0.35) [0.15–1.37]	0.28 (0.31) [0.04–2.00]	8 over 39 (21%)

NA, not available. ^a^Estimated data through the graph; ^b^Excluded case: did not meet inclusion criteria; ^c^Mean determinations of five consecutive days; ^d^Patient took 1,500 mg every other day alternating with 1,200 every other day; ^e^False elevation of blood lithium levels caused by contamination in a lithium heparin container commented by the author. ^f^Undetectable. ^g^Normal thyroid-stimulating hormone (TSH) range: 1-30-day-old infants: 0.52-16.00 µU/ml; 1-month to 5-year-old-children: 0.55-7.10 µU/ml (Siparsky and Accurso, 2007).

#### Infant-Plasma Lithium Concentration (I)

The mean infant plasma/serum lithium concentration was 0.23 (0.26) mEq/L (0.03–1.40).

#### Milk-to-Maternal Plasma Lithium Concentration Ratio (M/P Ratio)

The mean breast milk lithium concentration was 0.34 (0.14) mEq/L (0.10–0.69). The milk-to-maternal serum lithium ratio (M/P) was calculated in 28 samples. The average M/P ratio was 0.49 (0.19) (0.17–1.07). These data were obtained from a single time point.

#### Relative Infant Dose (RID)

Moretti et al. (2003)’s study was the only one to report the RID in infants exposed to lithium. The mean RID value was 12.2% (8.5%) (0–30%; median 11.2%; 95% CI, 6.3 to 18.0%); however, 11 of the 23 samples had a RID value between 10 and 25%, and 1 above 25%.

#### Infant-Plasma-to-Maternal Plasma Lithium Concentration Ratio (I/P Ratio)

The mean I/P ratio was 0.28 (0.31) (0.04–2.00). The I/P ratio was obtained from 47 samples.

Finally, Viguera et al. (2007) have described in her study a different index called the Infant-plasma-to milk lithium concentration ratio (I/M). The mean I/M ratio was 0.49 (0.35) (0.15-1.37).

### Clinical Adverse Effects in Breastfed Infants

Of the 39 breastfed infants included in this review, 8 (20.5%) showed a clinical adverse event. Two had transient lithium toxicity that recovered after discontinuation of breastfeeding (Tunnessen and Hertz, 1972; Skausing and Shou, 1977). Two cases had mild hypotonia, one for the first 2 days of life (Sykes et al., 1976), and the other after 2 months (Bogen et al., 2012). Finally, two cases of weight loss in the first week were recorded (Bogen et al., 2012), and one case with transient hypothyroid (increased TSH) and two cases with renal parameter alterations (increased creatinine and/or urea nitrogen parameters) (Viguera et al., 2007). Moreover, 2 of the 39 breastfed infants had congenital malformations (a congenital heart disease which underwent surgery on postpartum day 3 (Tanaka et al., 2008), and one hypospadias and right cryptorchidism (Marín et al., 2011) not related to the transfer of lithium during lactation. The case with congenital heart disease was initially suspected of having an acute transient lithium intoxication; however, authors explained that it was a false elevation of infant lithemia due to a contamination of lithium, heparin container. Finally, it must be said that four infants were exposed prenatally to lithium polytherapy (Tunnessen and Hertz, 1972; Bogen et al., 2012), and three during lactation (Sykes et al., 1976; Marín et al., 2011; Frew, 2015) (see **Tables 1** and **2**).

### Quality Assessment

**Table 3** shows the results of the quality assessment. Only 2 studies of the 13 included (15%) in the systematic review complied with 50% or higher of the quality check list items.

**Table 3:** Quality checklist of clinical lactation studies of the included studies based upon the ILCA, FDA, and EMA guidelines.

Author year	Weinstein and Goldfield, 1969	Fries, 1970	Tunnessen and Hertz, 1972	Schou and Amdisen, 1973	Sykes et al., 1976	Skausing and Shou, 1977	Montgomery, 1997	Moretti et al., 2003	Viguera et al., 2007	Tanaka et al., 2008	Marín et al., 2011	Bogen et al., 2012	Frew, 2015	
**Study design**														
Mother–infant pair design	Yes	Yes	Yes	Yes	Yes	Yes	Yes	Yes 2/11	Yes	Yes	Yes	Yes	Yes	100%
Other consideration: longitudinal	No	No	No	No	No	No	No	Yes	Yes	No	Yes	Yes	Yes	38%
Monotherapy/polytherapy	NA	NA	Yes	No	Yes	NA	NA	NA	Yes	NA	Yes	Yes	Yes	46%
**Clinical conduct**														
Clear sampling strategy	No	No	No	No	No	No	No	No	No	No	No	No	No	0%
Lithium dose	Yes	Yes	Yes	NA	Yes	NA	Yes	Yes	Yes	Yes	Yes	Yes	Yes	85%
Lithium frequency	NA	NA	NA	NA	NA	NA	NA	NA	NA	NA	NA	NA	NA	0%
Steady state	NA	NA	NA	NA	NA	NA	NA	NA	NA	NA	NA	NA	NA	0%
Time of drug intake until sampling	NA	NA	NA	NA	NA	NA	NA	NA	Yes	NA	NA	NA	NA	7%
Sampling at least in two of three matrices^a^	Yes	Yes	Yes	Yes 3/5	Yes	Yes	Yes	Yes 2/11	Yes 9/10	Yes	Yes	Yes	Yes	100%
Sampling assessed simultaneously	NA	NA	NA	NA	Yes	NA	NA	NA	Yes	Yes	Yes	Yes	Yes	46%
**Pharmacokinetic endpoints**														
Infant plasma/serum concentration	Yes	Yes	Yes	Yes 4/5	Yes	Yes	Yes	Yes 3/11	Yes	Yes	Yes	Yes	Yes	100%
Milk/plasma/serum ratio	Yes	Yes	Yes	Yes 4/5	Yes	No	No	No	Yes	Yes 1/2	No	No	No	54%
RID	NA	NA	NA	NA	NA	NA	NA	Yes 9/11	NA	NA	NA	NA	NA	7%
I/P ratio	Yes	Yes	Yes	Yes 3/5	Yes	Yes	Yes	Yes 2/11	Yes	No	Yes	Yes	Yes	92%
**Infant clinical monitorization**														
Adverse effect evaluated	Yes	Yes	Yes	NA	Yes	Yes	Yes	Yes	Yes	Yes	Yes	Yes	Yes	92%
Use of pediatric rating scale/systematic clinical evaluation	NA	NA	NA	NA	NA	NA	NA	NA	NA	NA	NA	NA	NA	0%
**Laboratory methods** **^b^**														
Methods description	NA	NA	NA	NA	NA	NA	NA	NA	NA	NA	NA	NA	NA	0%
Separate milk validation report	NA	NA	NA	NA	NA	NA	NA	Yes	NA	NA	NA	NA	NA	7%
Assay sensitivity reported	NA	NA	NA	NA	NA	NA	NA	NA	NA	NA	NA	Yes	NA	7%
Detection methods used reported	NA	NA	NA	Yes	NA	NA	NA	NA	Yes	NA	NA	Yes	NA	23%
**Total quality percentage of items (%)**	35%	35%	40%	30%	45%	25%	30%	45% ^c^	60%	35%	45%	55%	45%	

I/P ratio, infant to maternal plasma/serum lithium concentration ratio; M/P ratio, milk-to-maternal plasma ratio; NA, not available; RID, relative infant dose. ^a^Matrices: Maternal plasma/serum, milk, infant plasma/serum. ^b^Laboratory Technique: Schou and Amdisen (1973) Flame photometric method; Viguera et al. (2007) Lithium concentration in maternal and infant sera by standard commercial laboratory methods. Lithium concentration in breast milk by ion-selective electrode detection (Beckman Coulter, Fullerton, Calif.); Tanaka et al. (2008) ion especific electrode method on the Roche Integra 400 Analizer; Bogen et al. (2012) Inductively coupled plasma-mass spectrometry (quality control CVs of 4.0% to 7.0% at target levels of 0.025 and 1.800 meq/L with a lower limit of 0.01 meq/L. ^c^ Moretti et al. (2003) partially complies with 45% of the items (3/11).

All studies except Moretti et al. (2003) had a mother–infant pair design (Weinstein and Goldfield, 1969; Fries, 1970; Tunnessen and Hertz, 1972; Schou and Amdisen, 1973; Sykes et al., 1976; Skausing and Shou, 1977; Montgomery, 1997; Viguera et al., 2007; Tanaka et al., 2008; Marín et al., 2011; Bogen et al., 2012; Frew, 2015). Moretti et al. (2003) used a mixed design: in two cases, a mother–infant pair, and in nine cases, a lactating-women-milk-only design. Five studies had a longitudinal design (Moretti et al., 2003; Viguera et al., 2007; Marín et al., 2011; Bogen et al., 2012; Frew, 2015). As all studies included in the review were case reports or case series applying a clinical approach, none had a clear strategy for sample extraction. The maternal lithium dose during lactation was reported in 10 studies (Weinstein and Goldfield, 1969; Fries, 1970; Sykes et al., 1976; Moretti et al., 2003; Viguera et al., 2007; Tanaka et al., 2008; Marín et al., 2011; Bogen et al., 2012; Frew, 2015). No information on the frequency of lithium prescription and time of lithium intake until sampling was available in any of the studies. Seven out of the 13 studies had samples from all 3 matrices (mother and infant plasma/serum and milk) (Weinstein and Goldfield, 1969; Fries, 1970; Tunnessen and Hertz, 1972; Schou and Amdisen, 1973; Sykes et al., 1976; Viguera et al., 2007; Tanaka et al., 2008). Only one study stated specifically that sampling was obtained simultaneously in the three matrices (Viguera et al., 2007). Finally, none of the studies reported that sampling was taken in steady state.

With respect to pharmacokinetic end-points assessed, *the infant-plasma-lithium concentration (I)* was obtained in almost all cases (58 samples; 38/39 cases). Only 1 study (Viguera et al., 2007) assessed *the milk-to-mother plasma ratio (M/P)* in 10 women, but 6 more studies provided data for calculating this ratio (Weinstein and Goldfield, 1969; Fries, 1970; Tunnessen and Hertz, 1972; Schou and Amdisen, 1973, Sykes et al., 1976; Tanaka et al., 2008). In all cases, the M/P ratio was obtained from a single time point. The *RID* was calculated only in 1 study of the 13 (Moretti et al., 2003). In 7 of the 11 cases, milk was obtained from multiple samples at a specific dose interval. Finally, *the infant-plasma-to mother-plasma ratio (I/P ratio)* was studied in 12 studies (Weinstein and Goldfield, 1969; Fries, 1970; Tunnessen and Hertz, 1972; Schou and Amdisen, 1973; Sykes et al., 1976; Skausing and Shou, 1977; Montgomery, 1997; Moretti et al., 2003; Viguera et al., 2007; Marín et al., 2011; Bogen et al., 2012; Frew, 2015). Only four studies provided partial information on the laboratory methods used in the lithium measurement in plasma/serum and milk (Schou and Amdisen, 1973; Moretti et al., 2003; Viguera et al., 2007; Bogen et al., 2012).

Of the 13 studies, only 1 (Schou and Amdisen, 1973) did not report short-term infant adverse effects during lactation.

## Discussion

Breastfeeding is known to have clear general health benefits for mother and infant (WHO, 2017). However, delivery is a situation of biological and psychosocial stress, especially for vulnerable women (Sanjuan et al., 2008). Postpartum is a high-risk period for the initiation or recurrence of affective disorders (Munk-Olsen et al., 2009; Bergink et al., 2012). In recent years, interest has increased in supporting breastfeeding in women who may benefit from initiating or maintaining psychopharmacological treatment in the postpartum period. Lithium is considered a first-line treatment in bipolar disorder in most international guidelines (Malhi et al., 2017). In this systematic review, we assessed the current evidence and quality of studies that have evaluated lithium transfer to lactating infants, and their short-term outcomes.

This systematic review included data from 13 highly heterogeneous studies (39 cases); all of them case are reports or case series. The cases were informative, but the absence of a standard protocol makes interpretation difficult (Wang et al., 2017). This review is not without limitations. The search was restricted to PubMed and LactMed databases and to English and Spanish language peer-reviewed journals, and potentially we could miss studies published in other languages or in specific journals. The sample size, the degree of level of evidence, and quality of studies included were all less than optimal. Fewer than 16% of studies applied more than 50% of quality check-list items (see **Table 3**).

First of all, some of the studies included failed to report important variables that may have affected lithium concentration during lactation (**Table 1**): maternal factors (ethnicity, age, gravity/parity, weight, clinical diagnosis), pharmacokinetic factors [diet, smoking, alcohol intake, concomitant medication (i.e., non-steroidal anti-inflammatory drugs, angiotensin converting enzyme inhibitors, or diuretics as they may increase lithium concentration)] or other medical conditions (hyperemesis gravidarum, thyroid and renal illness, polyhydramnios, preeclampsia), and infant factors such as age (as we note above), term/preterm birth, and extended breastfeeding.

All studies included had a mother–infant pair design, but few were longitudinal. There were variations between maternal lithium dose and plasma/serum concentrations in mother, milk and infants, and also over the lactation period. We sought to establish whether there was a correlation between maternal and infant lithemia during the first week, but found very few data for this period (see **Table 2**). Moreover, as the majority of women had also been treated with lithium during late pregnancy, one might think that these initial infant lithemias were influenced more by placenta transfer than by lactation transfer. The milk-to-maternal plasma ratio (M/P) was below 1 in almost all cases (17/18). This indicated that lithium milk concentration was not superior to maternal concentration (i.e., there was no accumulation). With respect to the RID, studied only by Moretti et al. (2003), the results indicated that lithium can be used with caution during lactation. Although the RID is a well accepted measure of the safety of medication use during breastfeeding, as we noted above, it has substantial limitations (e.g., it does not take into account the age of the infant) (Anderson and Sauberan, 2016).

The pharmacokinetic parameter that provided the most information on infant safety was probably the infant-plasma concentration (Anderson and Sauberan, 2016). In the majority of the cases reviewed, the results were below 0.30 mEq/L, and no adverse effects were seen. However, six cases (Tunnessen and Hertz, 1972; Skausing and Shou, 1977; Montgomery, 1997; Moretti et al., 2003; Tanaka et al., 2008) showed higher lithemias (see **Table 2**). In the first case described by Tunnessen and Hertz, 1972, the infant developed cyanosis, hypothermia, hypotonia, and heart murmur within a few hours of birth. Infant lithemia was determined at 5 days of life when she experienced a cyanotic episode. At that time, the mother had a serum lithium concentration of 1.5 mEq/L, and the breast milk and the infant serum levels were 0.6 meq/L. The infant’s levels were completely normal by day 8. Her mother had taken lithium throughout pregnancy and the long-acting diuretic chlorthalidone prior to delivery, showing an increased lithemia (1.07–1.38 mEq/L). Since maternal and fetal lithium concentration are equal in utero, this intoxication might be due to a combination of maternal dose transfer and the slow renal excretion by the infant. It is known that levels of lithium clearance within the first days of delivery are a third of adult values after adjusting for differences in body surface area, but by the age of 6 months, this difference disappears (Lu and Rosembaum, 2014).

The second case, of Skausing and Shou (1977), was an infant who had been breastfeeding for 2 months without adverse effects, with an I/P ratio of 50%. At 2 months of life, after a respiratory infection and probably secondary dehydration, the infant showed signs of toxicity. Serum lithium levels were 1.4 mEq/L in the infant and 0.7 mEq/L in the mother, and the I/P ratio was 200%. The intoxication remitted after discontinuation of breastfeeding.

In the case reported by Tanaka et al. (2008) (see **Table 1**, case 33), the authors suspected that the toxic infant lithium level (4.19 meq/L) was spurious because of the absence of clinical symptoms of toxicity in the infant. Moreover, the other three cases with lithemia > than 0.30 mEq/L (Montgomery, 1997; Moretti et al., 2003) were not associated with adverse events. On the other hand, we found three cases with transient thyroid or renal parameters alterations in infants with lithemias between 0.23 and 0.10 mEq/L (Viguera et al., 2007). Lastly, there were two cases with weight loss in the first week of life with lithemias between 0.17 and 0.10 mEq/L published by Bogen et al. (2012). However, both cases were under polytherapy during pregnancy (antidepressant and lithium). These infants were born at term with adequate weight for gestational age, and both regained weight at 21 and 4 days, respectively, with breastfeeding support (Bogen et al., 2012). It seems that one of the most important variables for safety is infant age, related to changes in absorption, distribution, and excretion (Lu and Rosembaum, 2014). In this regard, adverse drug reactions occur in the first 2 months of life in close to 80% of cases exposed to drugs during lactation (Anderson et al., 2003; Soussan et al., 2014; Anderson et al., 2016). A similar figure was observed in the present review with lithium, supporting our results.

## Conclusions

The current information on lithium use during breastfeeding is based on a small and heterogeneous number of case reports and case series which have used different pharmacokinetic parameters of varying clinical relevance to estimate the short-term risk of lithium in nursing infants. In the studies included, 20% of infants presented transient short-term adverse effects.

The results of the review help us to identify several areas for improvement in future clinical research into lithium and lactation. Studies should include prospective longitudinal samples, recording a range of variables: socio-demographic, clinical (psychiatric, obstetric, and neonatal), therapeutic, and analytical; groups should be homogeneous (i.e., receiving monotherapy and/or polytherapy, with prenatal and/or postnatal lithium exposure); blood samples should be obtained simultaneously from mother–infant pairs, at several time points relative to delivery; useful pharmacokinetic parameters should be evaluated with validated laboratory methods; and a standardized clinical pediatric assessment of infants should be performed during lactation.

Based on the results of the systematic review, in the case of a woman who is reacting well to lithium therapy in the early postpartum period and chooses maternal lactation, we make the following recommendations:

- Multidisciplinary management in collaboration with obstetricians, pediatricians, toxicologists, and psychiatrists
- Prenatal discussion with the mother regarding the risk and benefits of breastfeeding with and without lithium
- In women treated with lithium during late pregnancy, monitoring and analysis of lithium levels in the mother–infant pair during delivery, at 48h postpartum and 10 days postpartum
- In women who initiate lithium treatment in postpartum, monitoring and analysis of lithium levels in the mother–infant pair at 10 days after starting treatment. Infant analysis should include thyroid and renal parameters.
- If infant lithemia is <0.30 mEq/L, lithemia monitoring should only continue in the mother–infant pair if there are clinical symptoms of lithium intoxication.
- Clinical monitoring of the infant should include weight gain, in addition to neurodevelopment. The mother should be referred to a breastfeeding support group or to an early intervention service if needed. Psychoeducation should be provided for parents or caregivers to monitor their infants for signs and symptoms of feeding problems, dehydration, hypotonia, and lethargy.

## Author Contributions

All authors had full access to all the data in the study and take responsibility for the integrity of the data and the accuracy of the data analysis. Case study concept and design: MI and RM-S. Acquisition, analysis, or interpretation of data: MI, MT and RM-S. Drafting of the manuscript: MI and RM-S. Critical revision of the manuscript for important intellectual content: All authors. Technical, or material support: MI and MT.

## Conflict of Interest Statement

In the last three years, RM-S has received grants/research support from the Instituto de Salud Carlos III, Spanish Ministery of Science, Innovation and University. 

The remaining authors declare that the research was conducted in the absence of any commercial or financial relationships that could be construed as a potential conflict of interest.
