# Supplementary material for: Clinical Lactation Studies of Lithium: A Systematic Review
Source: Front Pharmacol. 2019 Sep 10;10:1005. doi: 10.3389/fphar.2019.01005 (PMC6746934; doi:10.3389/fphar.2019.01005)
Supplement: Supplementary file 1 [file Table_1.docx]

**Supporting Information**

**Title:** Clinical lactation studies of lithium: a systematic review

**List of Supporting Material for the article**

Section I PRISMA guidelines checklist

Section II Excluded Studies

**I. PRISMA guidelines**

| Section/Topic Checklist item | | | |
| --- | --- | --- | --- |
| **TITLE** | ♯ |  | pp |
| Title | 1 | Identify the report as a systematic review, meta-analysis, or both. | 1 |
| **ABSTRACT** |  |  |  |
| Structured summary | 2 | Provide a structured summary including, as applicable: background; objetives; data sources; study eligibility criteria, participants, and interventions; study appraisal and synthesis methods; results; limitations; conclusions and implications on key findings; systematic review registration number. | 2,3 |
| **INTRODUCTION** |  |  |  |
| Rationale | 3 | Describe the rationale for the review in the context of what is already known. | 4-7 |
| Objetives | 4 | Provide an explicit statement of questions being adressed with reference to participants, interventions, comparisons, outcomes, and study design (PICOS). | 8 |
| **METHODS** |  |  |  |
| Protocol and registration | 5 | Indicate if a review protocol exists. If and where it can be accessed (e.g. Web address), and, if available, provide registration information including registration number. | 8 |
| Eligibility criteria | 6 | Specify study characteristics (e.g.PICOS, length of follow-up) and report characteristics (e.g. years considered, language, publication status) used as criteria for eligibility, giving rationale. | 8,9 |
| Information sources | 7 | Describe all information sources(e.g. databases with dates of coverage, contact with study authors to identify additional studies) in the search and date last searched. | 9 |
| Search | 8 | Present full electronic search strategy for at least one database, incluiding any limits used, such that it could be repeated. | 8,T1 |
| Study selection | 9 | State the process for selecting studies (i.e., screening, eligibility, included in systematic review, and, if applicable, included in the meta-analysis). | 9 |
| Data collection process | 10 | Describe method of data extraction from reports (e.g.piloted forms, independently, in duplicate) and any processess for obtaining and confirming data from investigators. | 8 |
| Data items | 11 | List and define all variables for which data were sought (e,g. PICOS, funding sources) and any asumptions and simplifications made. | 8,9 |
| Risk of bias in individual studies | 12 | Describe methods used for assessing risk of bias of individual studies (incluiding specification of whether this was done at the study or outcome level), and how this information is to be used in any data synthesis. | 13 |
| Summary measures | 13 | State the principal summary measures (e.g. risk ratio, difference in means). | 9,10 |
| Synthesis of results | 14 | Describe the methods of handling data and combining results of studies, if done, incluiding measures of consistency (e.g. I^2^) for each meta-analysis. | NA |
| Risk of bias across studies | 15 | Specify any assessment of risk of bias that may affect the cumulative evidence (e.g. publication bias, selective reporting withing studies). | T3 |
| Additional analyses | 16 | Describe methods of additional analyses (e.g. sensitivity or subgroup analyses, meta-regression), if done. Indicating which were pre-specified. | NA |
| **RESULTS** |  |  |  |
| Study selection | 17 | Give numbers of studies screened, assessed for elegibility, and included in the review, with reasons for exclusions at each stage, ideally with a flow diagram. | 13,F2 |
| Study characteristics | 18 | For each study, present characteristics for which data were extracted (e.g. study size, PICOS, follow-up period) and provide the citations. | T1/2 |
| Risk of bias within studies | 19 | Present data on risk of bias of each study and, if available, any outcome-level assessment (see ítem 12). | T1/2 |
| Results of individual studies | 20 | For all outcomes considered (benefits or harms), present, for each study: (a) simple summary data for each intervention group and (b) effect estimates and confidence intervals, ideally with a forest plot. | T2/3 |
| Synthesis of results | 21 | Present results of each meta-analysis done, including confidence intervals and measures of consistency. | NA |
| Risk of bias across studies | 22 | Present results of any assessment of risk of bias across studies (see ítem 15) | T3 |
| Additional analysis | 23 | Give results of additional analyses, if done (e.g. sensitivity or subgroup analyses, meta-regression (see ítem 16). | NA |
| **DISCUSSION** |  |  |  |
| Summary of evidence | 24 | Summarize the main findings including the strength of evidence for each main outcome: consider their relevance to key groups (e,g, health care providers, users, and policy makers). | 17-18 |
| Limitations | 25 | Discuss limitations at study and outcome level (e.g. risk of bias), and the review level (e.g. incomplete retrieval of identified research, reporting bias). | 18,19 |
| Conclusions | 26 | Provide a general interpretation of the results in the context of other evidence, and implications for future research. | 20-22 |
| **FUNDING** |  |  |  |
|  | 27 | Describe sources of funding for the systematic review and other support (e.g. supply of data): role of funders for the systematic review. | 23 |
| doi:10.1371/journal.pmed.1000100.t001 | | | |

**II. List of excluded papers and reasons**

**1. Of no interest with the aim of the review (N=**9)

-Rosso G, Albert U, Di Salvo G, Scatà M, Todros T, Maina G. Lithium prophylaxis during pregnancy and the postpartum period in women with lithium-responsive bipolar I disorder. *Arch Womens Ment Health* 2016;**19**:429-32.

-Frayne J, Nguyen T, Kohan R, De Felice N, Rampono J. The comprehensive management of pregnant women with major mood disorders: a case study involving phenelzine, lithium, and quetiapine. *Arch Womens Ment Health* 2014;17:73-5.

-Cohen LS, Sichel DA, Robertson LM, Heckscher E, Rosenbaum JF. Postpartum prophylaxis for women with bipolar disorder. *Am J Psychiatry* 1995;**152**:1641-5.

-Austin MP. Puerperal affective psychosis: is there a case for lithium prophylaxis? *Br J Psychiatry* 1992;**161**:692-4.

-Pinelli JM, Symington AJ, Cunningham KA, Paes BA. Case report and review of the perinatal implications of maternal lithium use. *Am J Obstet Gynecol* 2002;**187**:245-9.

-Malzacher A, Engler H, Drack G, Kind C. Lethargy in a newborn: lithium toxicity or lab error? *J Perinat Med* 2003;**31**:340-2.

-Stothers JK, Wilson DW, Royston N. Lithium toxicity in the newborn. *Br Med J* 1973; **3**:233-4.

-Morrell P, Sutherland GR, Buamah PK, Oo M, Bain HH. Lithium toxicity in a neonate. *Arch Dis Child* 1983;**58**:539-41.

-Wesseloo R, Wierdsma AI, van Kamp IL, Munk-Olsen T, Hoogendijk WJG, Kushner SA, et al. Lithium dosing strategies during pregnancy and the postpartum period. *Br J Psychiatry* 2017;**211**:31-6.

**2. Review papers, editorials or comments (N=**12)

-Wichman CL. Managing Your Own Mood Lability: Use of Mood Stabilizers and Antipsychotics in Pregnancy. *Curr Psychiatry Rep* 2016;**18**:1. doi: 10.1007/s11920-015-0646-1.

-Nielsen RE, Damkier P. Pharmacological treatment of unipolar depression during pregnancy and breast-feeding--a clinical overview. *Nord J Psychiatry* 2012;**66**:159-66.

-Kozma C1. Neonatal toxicity and transient neurodevelopmental deficits following prenatal exposure to lithium: Another clinical report and a review of the literature. *Am J Med Genet A* 2005;**132**:441-44.

-Thomas P, Severus WE. Managing bipolar disorder during pregnancy and lactation: is there a safe and effective option? *Eur Psychiatry* 2003;**18** Suppl 1:3s-8s.

-Llewellyn A, Stowe ZN, Strader JR Jr. The use of lithium and management of women with bipolar disorder during pregnancy and lactation. *J Clin Psychiatry* 1998;59 Suppl 6:57-64.

-Pons G, Rey E, Matheson I. Excretion of psychoactive drugs into breast milk. Pharmacokinetic principles and recommendations. *Clin Pharmacokinet* 1994;**27**:270-89.

-Schou M. Lithium treatment during pregnancy, delivery, and lactation: an update. *J Clin Psychiatry* 1990;**51**:410-3.

-Robinson GE, Stewart DE. Postpartum psychiatric disorders. *CMAJ* 1986;**134**:31-7.

-Linden S, Rich CL. The use of lithium during pregnancy and lactation. *J Clin Psychiatry* 1983;**44**:358-61.

-Dodd S, Berk M. The safety of medications for the treatment of bipolar disorder during pregnancy and the puerperium. *Curr Drug Saf* 2006;**1**:25-33.

-Stevens D, Burman D, Midwinter A. Lithium toxicity in the newborn. *Arch* *Dis Child* 1983;**58**:840.

-Galbally M, Bergink V, Vigod SN, Buist A, Boyce P, Chandra P, et al. Breastfeeding and lithium: is breast always best'. *Lancet Psychiatry* 2018;**5**:534-6.

**3. Pharmacokinetic data only in a single matrix (N=**2)

-Wise MG, Javors MA, Funderburg LG, Sabaratnam MS, Bowden CL. Lithium levels in bodily fluids of a nursing mother and infant. *Lithium* 1990;**1**:189-91.

-Marin Gabriel Ma, Malanana Martinez AM, Olza Fernandez I. Lithium while breastfeeding: report. *Arch Argent Pediatr* 2018;**116**:e319-e21.

**4. No pharmacokinetic date of interest (N=**6)

-Moretti ME, Lee A, Ito S. Which drugs are contraindicated during breastfeeding? Practice guidelines. *Can Fam Physician* 2000;**46**:1753-7.

-Koren G, Moretti M, Ito S. Continuing drug therapy while breastfeeding. Part 2. Common misconceptions of physicians. *Can Fam Physician* 1999;**45**:1173-5.

-Suri RA, Altshuler LL, Burt VK, Hendrick VC.Managing psychiatric medications in the breast-feeding woman. *Medscape Womens Health* 1998;**3**:1.

-Schou M, Weinstein MR. Problems of lithium maintenance treatment during pregnancy, delivery and lactation. *Agressologie* 1980;**21**:7-9.

-Stewart DE, Klompenhouwer JL, Kendell RE, van Hulst AM. Prophylactic lithium in puerperal psychosis. The experience of three centres. *Br J Psychiatry* 1991;**158**:393-7.

-Zegers B, Andriessen P. Maternal lithium therapy and neonatal morbidity. *Eur J Pediatr* 2003;**162**:348-9.
